# Oncology Nursing Minimum Data Set (ONMDS): can we hypothesize a set of prevalent Nursing Sensitive Outcomes (NSO) in cancer patients?

**DOI:** 10.3332/ecancer.2013.345

**Published:** 2013-09-02

**Authors:** A Milani, S Mauri, S Gandini, G Magon

**Affiliations:** European Institute of Oncology, via Ripamonti 435, 20141 Milano, Italy

**Keywords:** cancer patient, oncology nursing, Nursing Minimum Data Set, ONMDS, Nursing Sensitive Outcome, NMDS, NSO, nursing record, documentation

## Abstract

**Background:**

The nursing minimum data set (NMDS) was created in 1977 in the United States to collect uniform standardised data that could be comparable among different nursing areas or patients. So far, in the literature, an NMDS in an oncology setting has not yet been described. Considering an oncology nursing minimum data set (ONMDS), which data could be chosen to define this tool regarding cancer patient care?

**Material and methods:**

At the European Institute of Oncology (IEO), 20 experienced oncology nurses representing surgical, medical, and critical areas participated in a nursing record working group. All nurses followed an educational course on NMDS, and they shared clinical experiences to find which data common among different areas could be useful to care. To identify these data, nurses considered three issues: what is nursing care for nurses in the IEO? What is the nurses’ responsibility in the IEO? What is the organisational nursing model in the IEO? Nurses in the IEO are autonomous in decision making and recognised by patients and by a multi-professional team; the organisational nursing model is primary nursing with patient-centred care. Nursing data must therefore show the quality and results of this care. With this in mind, the working group decided to orient the ONMDS toward nursing-sensitive outcomes (NSOs), meeting also with psychologists, physiotherapists, and dieticians. Nurses analysed Oncology Nursing Society outcomes, and through focus groups, experiential meetings, role playing, and case studies, they integrated them with other NSOs.

**Results:**

The ONMDS is composed of 49 NSOs recognised as the most common and frequent oncologic outcomes regardless of the treatment that the patient undergoes. These outcomes were clustered into 15 categories. The categories are: gastrointestinal outcomes, genitourinary outcomes, respiratory outcomes, skin outcomes, fluid and electrolyte balance outcomes, neurological outcomes, security, functional status, vascular access outcomes, nutritional status, pain, psychosocial discomfort, activities of daily living (ADL), instrumental activities daily living (IADL), and self-care outcomes.

**Conclusions:**

Efforts to identify an ONMDS based on NSOs allow us to develop an tool that can standardise language, assessment, and intervention, but overall could be used to measure nursing care. To evaluate these potentialities, the ONMDS was introduced into nursing records, and it was tested with a pre–post research study.

## Introduction

The results of healthcare are the sum of several elements that contribute to the promotion and maintenance of health of the individual and the community. Results attributable to nursing care are described in the literature as nursing-sensitive outcomes (NSOs) [1]. An NSO is a behaviour, condition, or a measurable perception of the patient or his family that is achieved through or is significantly influenced by nursing intervention. Since 1960, early studies that targeted the measurement of performance in relation to a given intervention began to be reported. These were the years in which healthcare costs were starting to be discussed, with the consequent need to demonstrate efficacy with respect to the performance that sometimes contained non-profit public spending [2]. In the 1980s, interest in the results of nursing care reached a turning point, and the new goal became to define, standardise, and validate a set of measurable and globally accepted outcomes. The most important contribution came from the University of Iowa, who created three classification systems: the North American Nursing Diagnosis Association (NANDA), Nursing Intervention Classification (NIC), and Nursing Outcomes Classification (NOC) [3, 4]. Other systems were subsequently created; the aim of all of these classification systems was not only to create a complete taxonomy to help the identification of what we know and what is yet to be discovered (grey areas) but also to offer a common standardised language to improve communication among colleagues and codification for research electronic databases. Classification systems have multiplied, and the main interest is to investigate NSOs because they are the final result of nursing care and allow the evaluation of the effectiveness of nursing care. The scientific literature used to define nursing outcomes as positive or negative. In the first case, nursing care keeps the patient safe from adverse events (e.g. infections), but the most mentioned nursing outcomes are pressure ulcers, falls, patients’ claims, mistakes in drugs’administration, length of stay, deep vein thrombosis, mortality [5, 6]. Negative outcomes can be related not only to a low ratio between nurses and patients but also to a poor knowledge about safety, hands hygiene, infections, and so on. When nurses work on self-care with the aim to improve patients’ abilities to take care of themselves, they produced positive care outcomes. Nurses are able to influence patients’ compliance. 

A nursing minimum data set (NMDS) is composed of essential nursing data defined as the specific information that is used on a regular basis by the most of nurses in different contexts and in all nursing care. NMDS was born in the United States in 1977 but has been developed with different characteristics to meet the needs of individual countries, expanding into different demographic, nursing, and organisational elements [7]. In the Netherlands, the NMDS includes 24 items on nursing diagnoses, 32 on the activities, and four on the outcomes, while in Belgium, there are 17 items divided into six groups with a prevalence on activities, probably because the focus is mainly on nursing workload [8]. P-NMDS is an example of NMDS internationally validated describing nursing practice in perioperative context through 28 outcomes, 74 nursing diagnoses, and 133 interventions. In 2007, Park showed that the use of P-NMDS compared with the previous documentation, mainly based on diary nursing, better demonstrated the nursing contribution to patient care. 

## Aims

The main aim of this study is to test the feasibility of an Oncology Nursing Minimum Data Set (ONMDS) based on NSOs. The secondary aims are to integrate the ONMDS in a nursing record implementing nursing plan and to tailor nursing care to the collected data, which are updated and modified on the basis of patient reassessment. 

## Methods

### Research design

The project consists of a pre–post study test. 

### Pre-test

In the first phase, 20 experienced oncology nurses representing surgical, medical, and critical areas participated in a nursing record working group. They formed a focus group to identify critical or positive aspects of the instrument of nursing record that was used. After the focus, all nurses received an educational course about NMDS and NSO, and they shared clinical experience to find which data common among areas could be useful to care. The pre-test also included an objective evaluation. A checklist based mainly on the standards of the Joint Commission International (JCI) was developed to evaluate 50 medical records in the medical area.These are the standards evaluated:

— Care of patient (COP): nursing care is planned within 24 h from admittance; nursing care is tailored using the collected data; nursing plan is updated and modified on the basis of patient reassessment.
— Assessment of patient (AOP): Patients’ needs are identified on the basis of nursing and medical assessment and they are registered; all patients underwent a screening of pain; the patient is subjected to revaluation in order to determine the response to treatment; the patient is subjected to revaluation in order to plan for continuity of care; the patient is subjected to revaluation at appropriate intervals depending on the treatment plan and identified needs.

After the pre-test, the working group worked 4 h per week from May 2010 to October 2010 to identify a set of the most frequent and common cancer patient outcomes among medical, surgical, and critical nursing areas. This set composed our ONMDS, which is translated into a new instrument for nursing record. 

From March 2011 to November 2012, all nurses working at the European Institute of Oncology (IEO) were educated on this new tool, and it was implemented in all clinical wards. The ONMDS was also presented and shared with physiotherapists, psychologists, clinicians, and dieticians. 

### Post-test

After three months of nurses working with the ONMDS in the medical area, we performed the post-test. Fifty newly completed nursing records were analysed with the same checklist used in the pre-test to obtain an objective evaluation. When this new tool is implemented in all departments for at least one year, we will be able to perform another focus group analysis to compare the subjective evaluation of the pre-test. 

## Results

Experienced nurses sharing their clinical experiences found 49 NSOs recognised as the most common and frequent oncology outcomes regardless of treatment that patients undergo. These outcomes, common among all areas, constitute the ONMDS. These outcomes were clustered into 15 categories: gastro-intestinal outcomes, genito-urinary outcomes, respiratory outcomes, skin outcomes, fluid and electrolyte balance outcomes, neurological outcomes, safety, functional status, vascular access outcomes, nutritional status, pain, psychosocial distress, activity daily living (ADL), instrumental activity daily living (IADL), and self-care outcomes. In Table 1, the ONMDS is reported. 

Several outcomes were defined in collaboration with other professionals; the psychological area has been explored with a group of psycho-oncologists to identify the most frequent and common outcomes that could become the essential data in regard to NMDS. ‘Psychosocial distress’ that includes all differentiations of psychological problems was chosen in this regard. It is assessed with an international validated measuring instrument, which allows the patient to give a name to his emotions. Physiotherapists and dieticians have assessed other outcomes included in the ONMDS that make an important contribution to both the evaluation and the management of the outcomes. The ONMDS was also shared with the clinical forensic health department to obtain full consent, and today, all cancer patients are evaluated and taken into care on the basis of ONMDS. 

The introduction of the ONMDS in clinical practice showed a significant improvement of the JCI standards in nursing records. In Table 2, we can see the number of completed nursing records that met the JCI standards before and after the introduction of the ONMDS. 

## Discussion

An NMDS is a structured information system designed to collect uniform, standardised data, which are the most used by the most nurses in different care settings. It has been developed internationally not only to obtain essential nursing data but also to increase the visibility of nursing in the healthcare system. NSO, in contrast, is a behaviour, condition, or a measurable perception of the patient or his family that is achieved through or is significantly influenced by nursing intervention. The Oncology Nursing Society identified nursing-sensitive patient outcomes as effects or consequences of nursing care. They result in changes in patients’ experience of symptoms, safety, functional status, psychological distress, and healthcare costs.This work shows the feasibility of establishing in an oncology setting a NMDS based on NSOs. We proposed a set of 49 NSOs that are the most frequent and common to all cancer patients regardless of the treatment that they have undergone.The results of the pre–post study show that working with this tool allows nurses to plan care and to survey patients by obtaining more quality data in nursing records.The ONMDS would also be an important tool to implement epidemiological, organisational research and to monitor quality of nursing care. 

The ONMDS allows a better understanding of cancer patients’complexity, identifying the most common and frequent outcomes in these patients regardless of the treatment to which they are subjected; all patients are assessed using the ONMDS, and the comparison of the admission assessment with the discharge assessment allows the evaluation of nursing impact on patients’ outcomes improving nursing interventions quality and safety and above all the survey of outcomes during the entire period of hospitalisation. We observed that it promotes a new way of collecting nursing data that can be used to perform epidemiology research on health priority needs. Sharing an ONMDS provides a standard for the description of NSOs using a common terminology among oncology nurses working in different areas (medical, surgical, and critical) and ensures a standardised and shared data source. As our results show, this tool lets nurses plan tailored nursing care that is updated and modified on the basis of outcomes evolution achieving a continuity of care. Nurses use the Common Terminology Criteria Adverse Event Scale to complete the ONMDS, and this also allows a standardised language shared among all professionals. The next step could be the sharing of the ONMDS among different oncology centres to obtain a National and International consensus on it to achieve an important widely used database useful for research. 

## Acknowledgments

We would like to thank Alvisa Palese for her important contribution to the training of personnel involved. We would like to thank all the nurses who allowed the elaboration and implementation in clinical practice of this tool, especially Carmen Beltrami, Veronica Brunelli, Anna Brunoldi, Michela Cerone, Gianluca Cherchi, Margherita Clerici, Emanuela D’Anna, Chiara Esposito, Cristina Grossi, Ester Spacca, Antonella Gandini, and Andrea Zanoni. 

## Figures and Tables

**Table 1:** ONMDS by European Oncology Institute.

Current status	NSO	
Gastro-intestinal	Dry mouth	Vomiting
	Mucositis oral	Diarrhoea
	Dysphagia	Constipation
	Anorexia	Abdominal distension
	Nausea	Faecal incontinence
Genito-urinary	Urinary incontinence	Urinary tract infection
	Urinary retention	Sexual dysfunction
Respiratory	Dyspnoea	
	Cough	
	Productive cough	
	Lung infection	
Skin	Skin ulceration	
	Burn	
	Wound complication	
	Wound infection	
Fluid and electrolyte balance	Localised oedema Dehydration	
Neurological		
Safety	Allergic reaction	
	Deep vein thrombosis	
	Falls	
	Chills	
Functional status	Fatigue	
	Body odour	
Vascular access	Extravasation	
	Phlebitis	
	Haematoma	
	Injection-site reaction	
	Infusion-related reaction	
	CRSBI	
Nutritional status	Weight loss	
	Glucose intolerance	
	Diet	
	Artificial nutrition	
Pain		
Psychosocial distress		
ADL/IADL		
Self-care	Treatment	
	Devices	
	Nutrition	
	Rehabilitation technique	
	Pain	

ONMDS, oncology nursing minimum data set; NSO, nursing-sensitive outcome; ADL, activity daily living; IADL, instrumental activity daily living.

**Table 2:** JCI standards satisfaction.

Standard JCI	Pre-test	Post-test	Total	*p*-value
	Total clinical records (50)	Total clinical records (50)		
Nursing care is planned within 24 h from admission (COP 2.1.a)	0 (0%)	50 (100%)	50	<.0001
Nursing care is tailored on collected data (COP 2.1.b)	0	36 (72%)	36	<.0001
Nursing plan is updated and modified on the basis of patient reassessment (COP 2.1.c)	0	48 (96%)	48	<.0001
Patients’ needs are identified on the basis of nursing and medical assessment and they are registered (AOP.1.3.b)	9 (18%)	50 (100%)	59	<.0001
All patients underwent a screening of pain (AOP.1.8.2.a)	48 (96%)	50 (100%)	98	0.4949
The patient is subjected to revaluation in order to determine the response to treatment (AOP.2.a)	10 (20%)	50 (100%)	60	<.0001
The patient is subjected to revaluation in order to plan for continuity of care (AOP.2.b)	0 (0%)	46 (92%)	46	<.0001
The patient is subjected to revaluation at appropriate intervals depending on the treatment plan and needs identified (AOP.2.c)	0 (0%)	44 (88%)	44	<.0001

JCI, Joint Commission International; COP, care of patient; AOP, assessment of patient.
